# Investigation on Enamel and Dentine of Tooth through 1D Photonic Structure to Identify the Caries in Human Teeth

**DOI:** 10.3390/bioengineering9120788

**Published:** 2022-12-09

**Authors:** Mohammad Khursheed Alam, Vigneswaran Dhasarathan, Moustafa H. Aly, Mahmud Uz Zaman, Kiran Kumar Ganji, Rehana Basri, Manay Srinivas Munisekhar, Anil Kumar Nagarajappa

**Affiliations:** 1 Preventive Dentistry Department, College of Dentistry, Jouf University, Sakaka 72345, Al-Jouf, Saudi Arabia; 2 Department of Electronics and Communication Engineering, Centre for IoT and AI (CITI), KPR Institute of Engineering and Technology, Coimbatore 641407, India; 3 Electronics and Communication Engineering Department, College of Engineering and Technology, Arab Academy for Science, Technology and Maritime Transport, Alexandria 1029, Egypt; 4 Oral and Maxillofacial Surgery and Diagnostic Sciences Department, College of Dentistry, Prince Sattam Bin Abdulaziz University, Al-Kharj 16245, Saudi Arabia; 5 Department of Internal Medicine, College of Medicine, Jouf University, Sakaka 72345, Al-Jouf, Saudi Arabia; 6 Department of Oral & Maxillofacial Surgery & Diagnostic Sciences, College of Dentistry, Jouf University, Sakaka 72345, Saudi Arabia

**Keywords:** enamel, dentine, 1D photonic structure, reflectance

## Abstract

In this research, a one-dimensional (1D) photonic structure was employed to study the nature of both enamel and dentine teeth at the signal of 1.8 THz. A simple three layer one-dimensional crystal is chosen to avoid fabrication intricacy. The materials and methods for sample preparations are discussed. The principle of investigation of caries in the teeth relies on the amount of reflected signal from the structure. Similarly, reflectance is a function of refractive indices and thickness of each layer, the nature of both substrate and infiltrated materials, and the configuration of the structure. Apart from this, the fabrication process of one-dimensional structure and experimental set-up was proposed in this article. The numerical treatment is explained here to obtain reflectance, and subsequently, the output potential. Comparison studies on output potential between enamel and dentine are also shown through graphical representation. The output result in terms of milli-Volt (mV) were obtained at the output end and collected at the photodiode. Interesting results were also observed at the photodetector. For example; the output potential of the reflected signal is around 0.18 mV for both enamel and dentine teeth whereas the potential is more than 0.26 mV and 0.31 mV for caries in dentine and enamel, respectively. Finally, it was inferred that the nature of teeth pertaining to the caries in the enamel and dentine teeth can be investigated by identifying the amount of potential at the output end.

## 1. Introduction

Dental enamel is the strongest and most mineralized dead tissue. Current vertebrate species provides maximal flexibility and allow teeth to act as tools, food processors and weapons. Enamel formation and mineralization is a complex process that is strictly controlled by ameloblasts, including cells of the enamel organ. The highly polarised cells form a monolayer around the growing enamel tissue and travel in predetermined paths as a unitary emerging face to establish a proteinaceous matrix that serves as a template for crystal formation [1]. In this one-of-a-kind case, ameloblasts manage crystal development in this specific environment by influencing mineral and ion transport, proteolysis, pH control and endocytosis, among other things. The bulk of the enamel tissue is created and then solidified by these same cells and retransformation of dimension and properties occurs in different vertebrates [2]. Under certain pH conditions, ameloblasts retain intercellular connections and form a semi-permeable barrier that takes nutrients and ions from blood vessels at one end (proximal/basal) and form extracellular crystals at the other end (distal/secretory/apical) [3]. The advancement in caries detection has the potential to significantly enhance oral health issues. Present caries finding rates indicate the development of enamel. This report provided an initial investigation of terahertz pulse imaging (TPI) applications for caries detection. TPI was used to detect initial time periods of caries through enamel layers of cross-section areas of human teeth [4].

Hypomineralised enamel displays distinct absorption spectrum as well as a gap of carious enamel in TPI pictures. Both early diagnosis and safety are critical for applying TPI, which has significant implications for the applications of medical imaging. There are currently no extremely sensitive and precise clinical methods for diagnosing early-stage caries [5]. Early identification of caries in enamel could be of major clinical value. To avoid operational intervention, reverse of the decay process is applied. Terahertz pulse imaging (TPI) is a comparatively recent imaging technology with applications in both nonbiological [6] and biological subjects [7]. The coherent detection system employs just microwatts of non-ionizing light. Because our system’s exposure levels are orders of magnitude lower than those seen in nature, our method will be safer than the employment of X-rays [8]. In addition, as a distinct radiography, TPI provides a range of distinct wavelengths for each measured pixel. This opens up the prospect of employing the spectrum for diagnostic purposes other than merely monitoring mineralization level. Many materials exhibit terahertz resonant absorptions, which can be utilised to recognize the material. There is a link of noticeable and recognisable absorption with optical transitions between distinct quantized states of molecular rotational motion in mixture analysis. At these frequencies, vibration and irrational modes linked with molecular nuclei in proteins. Furthermore, with the existence DNA, the conformational state of the molecules are allowed for evaluation [9]. When terahertz spectroscopic characteristics are exploited in a scanning system, it is feasible to create compositionally insightful pictures of a variety of materials. It allows the internal structure of multi-layered dielectrics for the investigation of teeth [10]. The TPI method is employed in terahertz detection technologies [11] and is enabled directly for the measurement of the terahertz pulse. This allows the observed terahertz pulse’s form to be precisely calculated in time. It enables the development of spectra, the measurement of essential material characteristics and result shows the determination of key diagnostic qualities. This study of the imaging modalities underwent terahertz, transmission and reflection modes to conduct tests and identify fundamental material characteristics [12].

### 1.1. Teeth

The study of teeth used a shared model, which was obtained across Scotland with the informed permission of all in compliance with relevant national and local Ethics Committee criteria. The vertical portions of the teeth examined were approximately 200 nm thick and included healthy enamel and dentine tissue areas as well as problematic regions. Two forms of defective enamel were imaged: fissure caries and hypomineralisation [13]. In the beginning, imaging of early stage caries was taken vertically through molar teeth and hemisections of premolars as well as molars; this allowed for correct recognition and optimization of the different gap mechanisms in TPI.

### 1.2. TPI System

The TPI system utilised an amplified ultrafast Ti:Sapphire system is to be generated 250× 10–15 s pulses with a wavelength centering on 800 nanometre. The pulse speed was 250 KHz with a power output of 750 milliWatts on average. The output of the laser was divided into two beams. One was for terahertz creation and the other for terahertz detection after the terahertz beam passed through the sample. Terahertz was generated by passing the generating beam through a ZnTe crystal [14]. This terahertz radiation was sent to the sample and captured before being focussed on. The detecting beam was collinearly routed through this crystal with terahertz [15]. The system’s practical terahertz bandwidth ranged from 0.5 to 2.7 THz. In transmission, attenuation of the spectrum was created by taking a reference power range without a present sample. Power spectrum was acquired by dividing the sample present in the rescaling of units [16].

### 1.3. Transmission

Transmission may be used to estimate the refractive index(ri) as well as absorption coefficients of enamel, dentine and cavities. The moist sample was enclosed in two polymer thin films to preserve hydration levels. Coatings modify and/or increase the functionality of bulk surface or substrate. The width of the coatings was far smaller than the wavelength of the terahertz and hence it would have a minor influence on the data, which were experimentally proved to be terahertz transparent. As far as similar types of investigations are concerned; the status of coronavirus can be realised using two dimensional photonic crystal structure using plane wave expansion method and FDTD method, respectively. It was also shown that the outcomes pertaining to PWE [17] and FDTD [18] found similar results. However, the proposed structures in these references are hard to design because of their complexity. To avoid the same issues, we, in this research, (aim of the work) considered a one-dimensional photonic structure to investigate the caries in the teeth. Amelogenesis is the process through which enamel is formed. Ameloblasts produce enamel proteins into the enamel space, which are then destroyed and proteolytically eliminated by ameloblasts. Ameloblasts govern the creation of a de novo hydroxyapatite inorganic material with great accuracy of enamel region [19].

The creation of enamel is made of rods formed by a single ameloblast and stretching. The dentino-enamel junction (DEJ) is the enamel surface and interrod enamel contiguous to the enamel rods. EMP peptide traces are formed from enamel, and are thought to contribute to the final structure: fully formed (mature) enamel with distinctive morphological and biomechanical qualities. Mature enamel is 1–2 percent organic material and it is composed of 95 percent mineral and 2–4 percent water [20,21,22,23,24,25]. The current research work is arranged as follows. Section 1 gives a summary of dental and other issues with respect to the caries. Section 2 gives the materials and methods including the proposed structure and the principle of an operational mechanism. Numerical expression for reflectance and output potential is discussed in Section 3. Results are shown in Section 4, and discussions are presented in Section 5. Conclusions are drawn in Section 6.

## 2. Materials & Methods and Structure & Mechanism

The variety of teeth considered in this research came from Scotland. These teeth are the members of enamel and dentine tissues, such that they are normal and abnormal.

**Figure 1:**
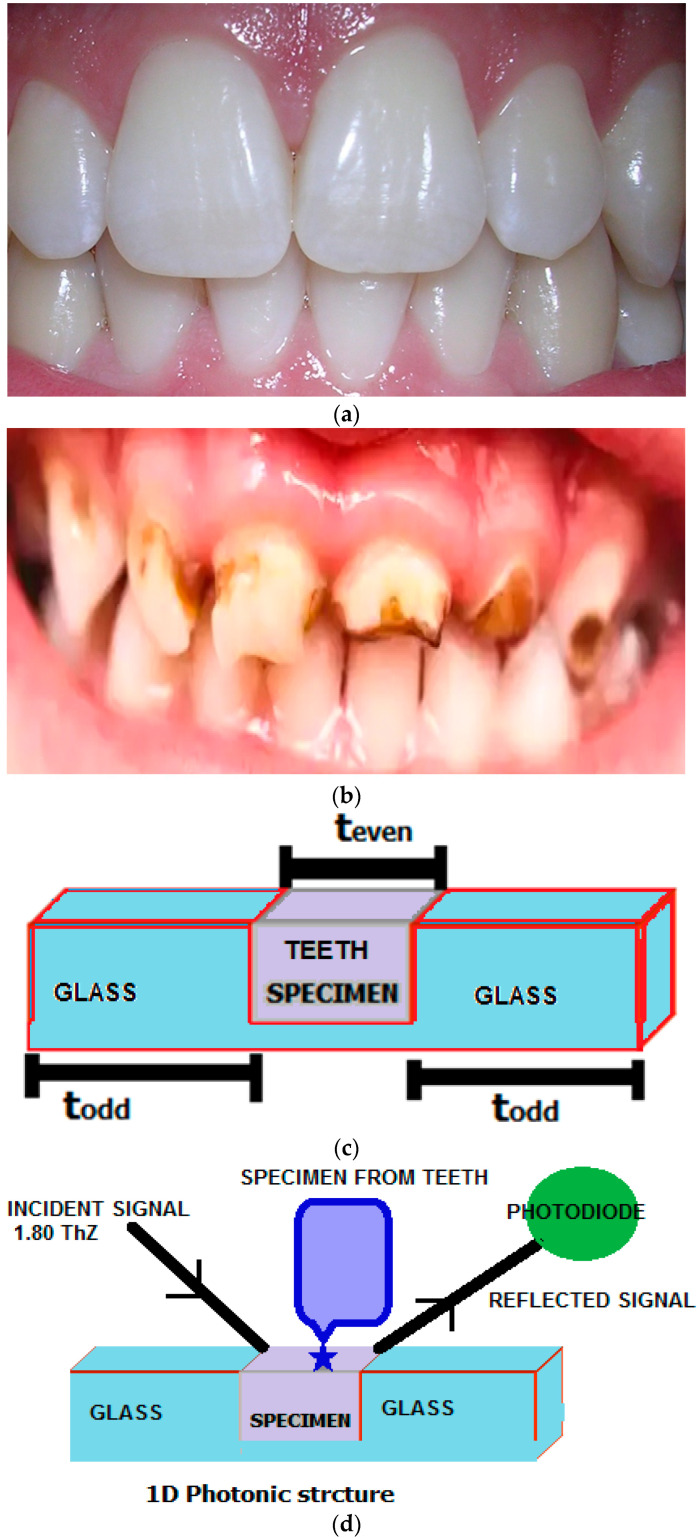
(**a**) Picture of normal teeth (no caries); (**b**) picture of abnormal teeth (caries); (**c**) one-dimensional photonic crystal structure to measure the specimen; and (**d**) the experimental setup to measure the deformation.

Here, the normal teeth (Figure 1a) of enamel and dentine indicates that it does not have any caries whereas abnormal (Figure 1b) enamels and dentine refers to the caries of in the teeth, For example the strength of caries can be realised as the amount of caries in the respective teeth. The refractive indices of these teeth were calculated with the help of an experimental set up and transmission system. The refractive index for different enamels and dentine are indicated in the Table 1.

Table 1 indicates the deviation of refractive indices for enamels and dentine teeth with respect to their caries. In this table (Table 1), the first column indicates the type of teeth and second column indicates the different types of enamels and dentine pertaining to the loads. Finally, column 3 specifies the refractive indices of the specimens. Moreover, it is also realised that the refractive indices are varied in a nonlinear manner. Again, it is clearly informed that A, B, C and D are nothing but the strength of caries in their respective teeth. Here the strength of caries is more for D and less in the case of A, where caries (D) > caries (C) > caries (B) > caries (A). Apart from this, the structure of the one-dimensional photonic structure and working principle plays a vital role in investigating the status of enamel and dentine teeth. Even though one-dimensional (1D) photonic crystal construction has consistently made an important contribution to the photonic society, the Figure 1c,d represent a one-dimensional photonic structure that is experimentally feasible to investigate the caries of teeth. As far as the layers of the structure are concerned, it consists of three layers, such that layers 1 and 3 are made up of glass materials, and the second layer consists of specimens of teeth. In this figure, todd and teven represent the widths of the odd and even layers, respectively, with thicknesses of 5 µm, 3 µm, and 5 µm for the first, second, and third layers, respectively. In terms of fabrication, references [26,27,28,29] detail the various studies conducted in relation to the experimental work. For example; Dou et al. provide an idea of the fabrication of a titanium metal oxide based one-dimensional photonic structure for a calorimetric sensor that measures volatile organic compounds and relative humidity [26]. Similarly, Shen et al. fabricated one-dimensional periodic nanostructures to examine the different chemical components [27]. One-dimensional photonic crystal was used to create erbium chloride silicate with nanowire [28]. Ilinykh et al. characterised a one-dimensional multilayer structure through visible–near IR spectroscopy, atomic force microscopy, and ellipsometry [29]. Aside from these, the reference (Gutierrez et al. 2019) shows a method to fabricate porous silicon (pSi)-based one-dimensional photonic crystals by photo acoustics for radiometry applications [30]. Again, Garca et al. use the physical vapour oblique angle deposition method to propose a TiO2/SiO2 based structure of a one-dimensional photonic crystal, and near infrared reflection (NIRR) images at 850 nm were employed for caries detection using 3D range data of teeth [31,32]. Because the current structure of a photonic crystal is similar to the reference discussed above, a glass-based, one-dimensional model for computing the amount of caries in a tooth could be created.

Aside from these studies, two research articles have recently been published in the references [33,34] relating to a one-dimensional photonic structure for biological sensors that measure alcohol concentration and salinity in water. Even though the authors considered a one-dimensional photonic structure, the defectiveness (in reference [33]) and clumsy structure led to complicated fabrication. Further focusing on the application of the present research, the proposed works could be used for biosensing applications through which the status of the teeth could be realised instantly. For example, whether the teeth are normal (no caries) or not (caries).

## 3. Numerical Expression for Reflectance and Output Potential

The output powers emerging from the photonic structure depend on the efficiency of the transmitted signal or the transmittance of the signal through the structure. Furthermore, the transmitted signal is a function of the reflection and absorption loss.

The reflectance of the 1D layer was analysed during the wave equation and was derived from Maxwell’s equation and subsequently through the Helmholtz equation. The wave equation, in terms of the second-order differential equation [15], can be written as:

(1) $$\frac{\partial^{2}E_{z}\left(x\right)}{\partial x^{2}}+n^{2}\left(x\right)\frac{\omega^{2}}{c^{2}}E_{z}\left(x\right)=0$$

where *E*, *ω*, *n*, and *c* are the electric field, frequency, refractive index and velocity of light respectively

(2) $$E_{j}\left(x\right)=A_{j}e^{i.n_{j}k.x_{j}}+B_{j}e^{-i.n_{j}k.x_{j}}$$

where *A* and *B* represent as the amplitudes of forward as well as backward waves.

But *x_j_* is the coordinate of *j*th interfaces, *k* can be represented the propagation constant (‘*ω*/*c*’). *A* (forward) and *B* (backward) of the signal is in the projected structure.

To determine *A* and *B*, the suitable condition at interfaces between the consecutive layers is applied and it is written as (electric field of function of ‘*x*’)

(3a) $$E_{j}=E_{j+1}$$

(3b) $$\frac{\partial E_{j}}{\partial x}=\frac{\partial E_{j+1}}{\partial x}$$

Adding Equation (2) as well as (3), we find that

(4a) $$A_{j}e^{i.n_{j}k.x_{j}}+B_{j}e^{-i.n_{j}k.x_{j}}=A_{j+1}e^{i.n_{j+1}k.x_{j}}+B_{j+1}e^{-i.n_{j+1}k.x_{j}}$$

(4b) $$i.n_{j}.k.A_{j}e^{i.n_{j}k.x_{j}}-i.n_{j}.k.B_{j}e^{-i.n_{j}k.x_{j}}=i.n_{j+1}.k.A_{j+1}e^{i.n_{j+1}k.x_{j}}-i.n_{j+1}.k.B_{j+1}e^{-i.n_{j}k.x_{j}}$$

The above equations can be written in form of matrix as

(5) $$\left(\begin{array}{cc} A_{0} & B_{0}\\ ikn_{0}A_{0} & -ikn_{0}B_{0} \end{array}\right)\left(\begin{array}{c} e^{ikn_{0}x_{0}}\\ e^{-ikn_{0}x_{0}} \end{array}\right)=\left(\begin{array}{cc} A_{1} & B_{1}\\ ikn_{1}A_{1} & -ikn_{1}B_{1} \end{array}\right)\left(\begin{array}{c} e^{ikn_{1}x_{0}}\\ e^{-ikn_{1}x_{0}} \end{array}\right)\;\left(\begin{array}{cc} A_{1} & B_{1}\\ ikn_{1}A_{1} & -ikn_{1}B_{1} \end{array}\right)\left(\begin{array}{c} e^{ikn_{1}x_{1}}\\ e^{-ikn_{1}x_{1}} \end{array}\right)=\left(\begin{array}{cc} A_{2} & B_{2}\\ ikn_{2}A_{2} & -ikn_{2}B_{2} \end{array}\right)\left(\begin{array}{c} e^{ikn_{2}x_{1}}\\ e^{-ikn_{2}x_{1}} \end{array}\right)\;\left(\begin{array}{cc} A_{2} & B_{2}\\ ikn_{2}A_{2} & -ikn_{2}B_{2} \end{array}\right)\left(\begin{array}{c} e^{ikn_{2}x_{2}}\\ e^{-ikn_{2}x_{2}} \end{array}\right)=\left(\begin{array}{cc} A_{3} & B_{3}\\ ikn_{3}A_{3} & -ikn_{3}B_{3} \end{array}\right)\left(\begin{array}{c} e^{ikn_{3}x_{2}}\\ e^{-ikn_{3}x_{3}} \end{array}\right)\;\left(\begin{array}{cc} A_{3} & B_{3}\\ ikn_{3}A_{3} & -ikn_{3}B_{3} \end{array}\right)\left(\begin{array}{c} e^{ikn_{3}x_{3}}\\ e^{-ikn_{3}x_{3}} \end{array}\right)=\left(\begin{array}{cc} A_{4} & B_{4}\\ ikn_{4}A_{4} & -ikn_{4}B_{4} \end{array}\right)\left(\begin{array}{c} e^{ikn_{4}x_{3}}\\ 1 \end{array}\right)$$

We solve the linear system equation by means of Crammer’s method. After obtaining the constants of *A* and *B*, putting these values in the Equations (2) and (3), the electric field at the output end is obtained. As far as the reflected signal is concerned, the reflectance of the signal (reflection loss) can be computed using the following expression:

(6) $$R={\left(1-E_{j}\right)}^{2}$$

Further the reflected energy can be written as

(7) $$\mathrm{Energy}\left(\mathrm{Reflected}\right)=R\times \mathrm{Energy}\left(\mathrm{incident}\right)$$

The output potential appeared at the photodiode is

(8) $$V=\frac{\mathrm{Energy}\left(\mathrm{Reflected}\right)}{\mathrm{Electronic}\;\mathrm{charge}\left(e\right)}$$

## 4. Results

**Figure 2:**
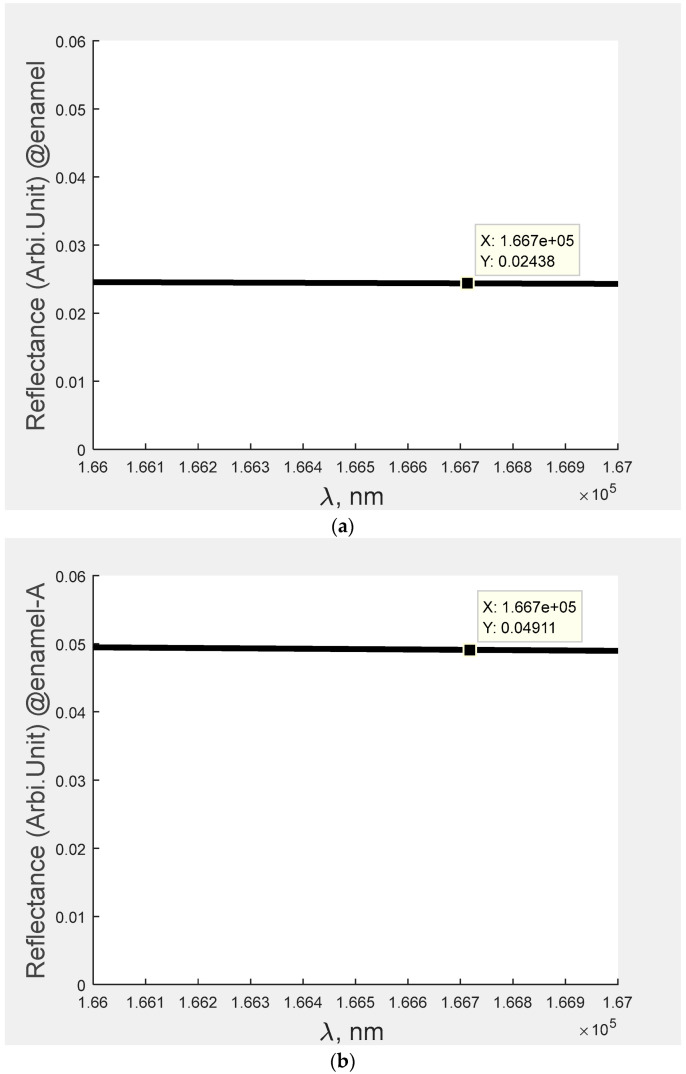

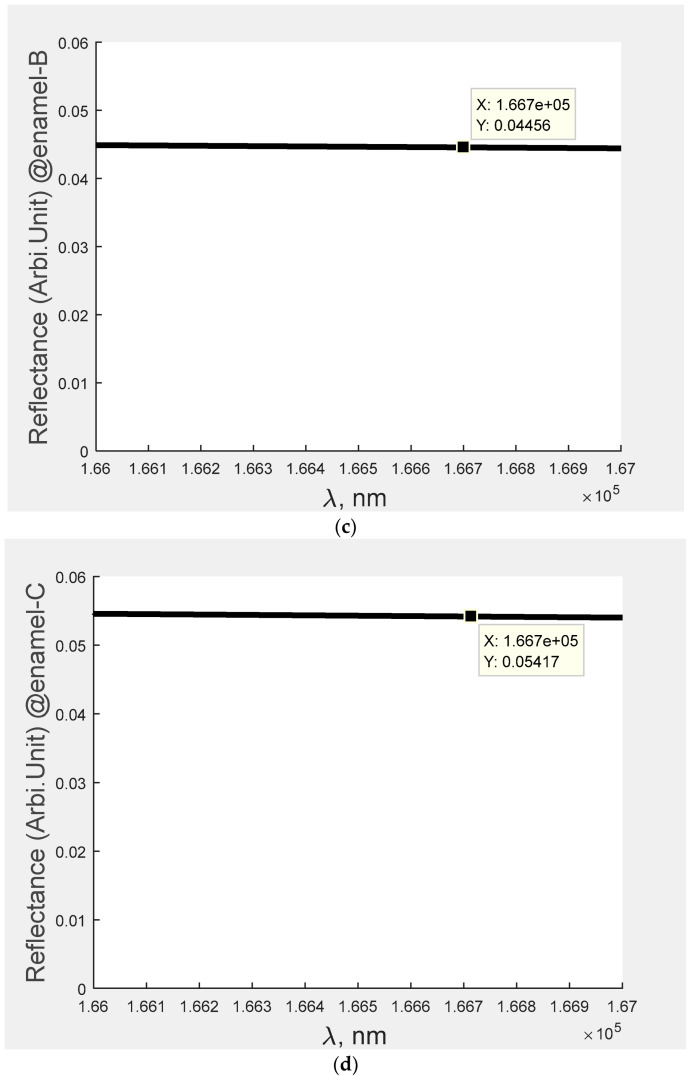

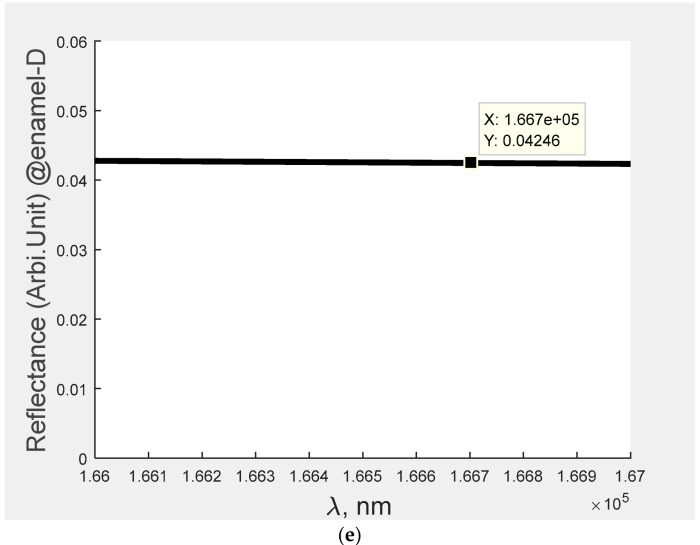
(**a**). Reflectance of enamel normal teeth at the signal 1.8 THz (frequency); (**b**) reflectance of enamel abnormal teeth (A) at the signal 1.8 THz (frequency); (**c**) reflectance of enamel abnormal teeth (B) at the signal 1.8 THz (frequency); (**d**) reflectance of enamel abnormal teeth (C) at the signal 1.8 THz (frequency); and (**e**) reflectance of enamel abnormal teeth (D) at the signal 1.8 THz (frequency).

**Figure 3:**
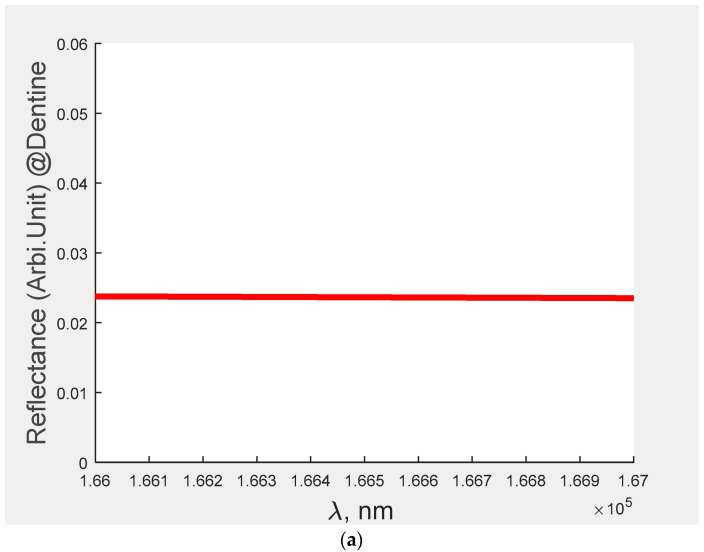

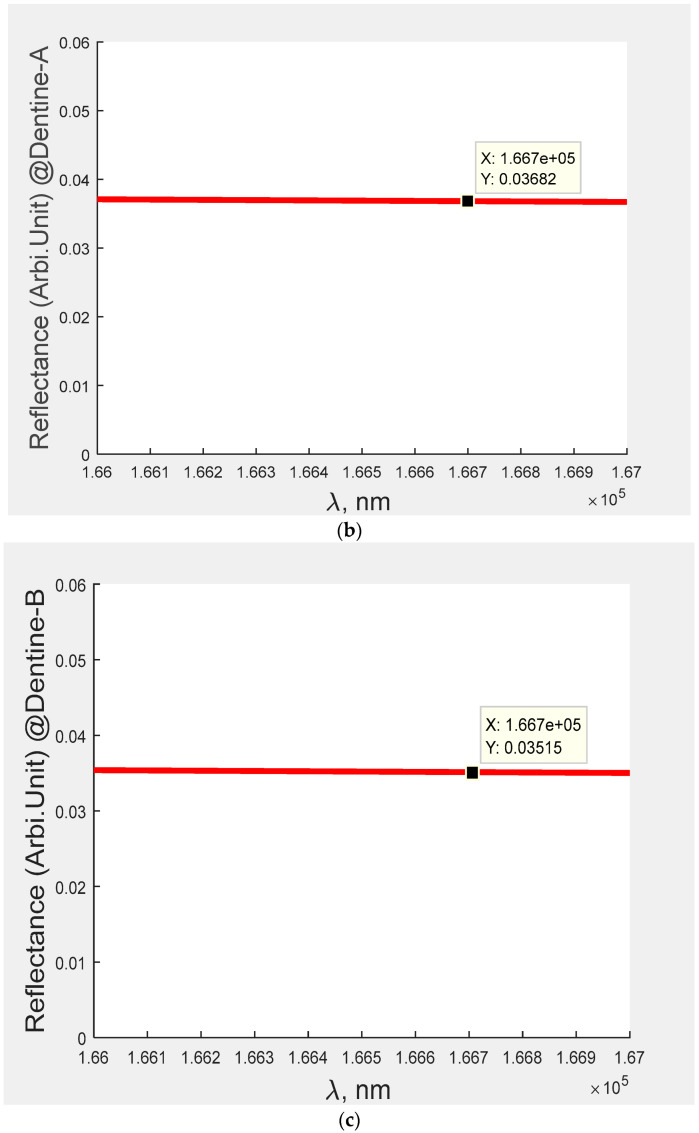

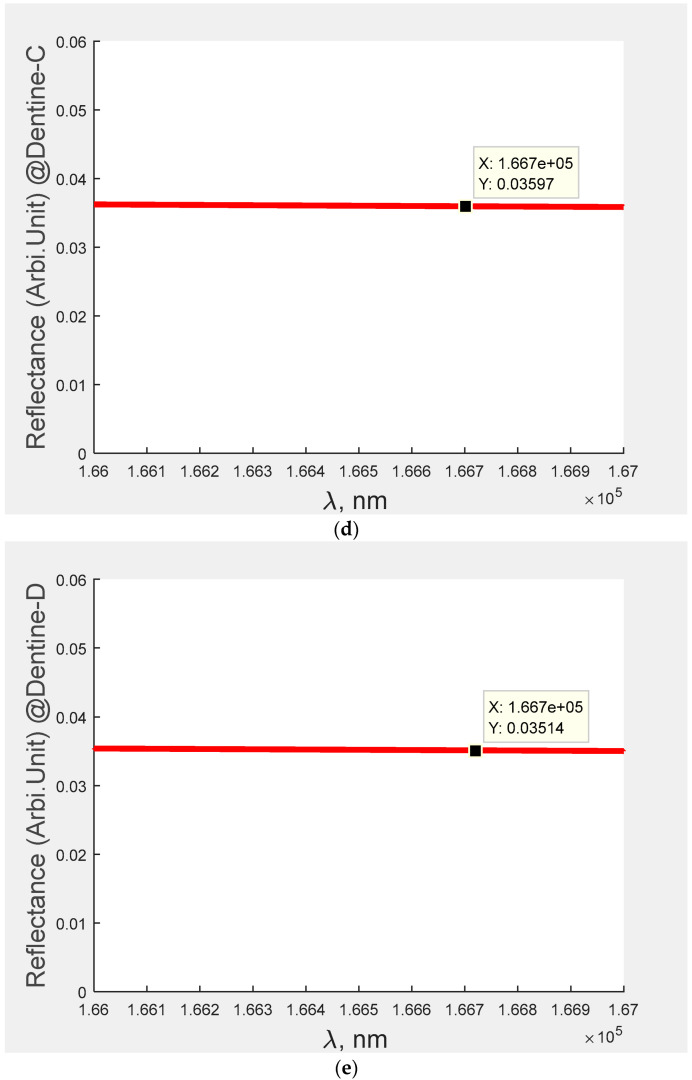
(**a**) Reflectance of dentine normal teeth at the signal 1.8 THz (frequency); (**b**) reflectance of dentine abnormal teeth (A) at the signal 1.8 THz (frequency); (**c**) reflectance of dentine abnormal teeth (B) at the signal 1.8 THz (frequency); (**d**) reflectance of enamel abnormal teeth (C) at the signal 1.8 THz (frequency); and (**e**) reflectance of enamel abnormal teeth (D) at the signal 1.8 THz (frequency).

From Section 3, it is understood that Figure 1c computes the amount of caries in the teeth at the signal of 1.8 THz. In this figure, when an incident signal falls in the three-layer structure having the specimen of teeth and subsequently some amount of the signal would be reflected. Furthermore, the reflected signal incidents on the detector converts its potential counterpart. The amount of reflected signal plays a significant role because it determines the amount of potential reflected at the photodiode that leads to estimation of caries in the teeth with respect to the enamel and dentine. Furthermore, with the help of Equations (1)–(6), the reflectance can be obtained by plane wave expansion. The results for the same are indicated in the Figure 2 and Figure 3 for enamel and dentine, respectively.

## 5. Discussion

Figure 2 and Figure 3 provide the information of the reflectance of different teeth with respect to their caries. For example, Figure 2a represents the reflectance pertaining to the no caries for enamel, whereas the reflectance curve for the Figure 2b–e indicates the specimen contains caries of different strengths.

Similarly, Figure 3a represents the reflectance pertaining to the no caries for dentine and reflectance curve for the Figure 3b–e indicates the specimen contains caries of different strengths. For example, in the abnormal teeth, A contains less caries as compared to the teeth B, C and D. Here Caries D > Caries C > Caries B > Caries A. After analysing the figure (Figure 2 and Figure 3), the reflectance for each specimen was computed and the same is indicated in each figure.

In Table 2, Column 3 and 4 represents the reflectance of signal with respect to the different caries of teeth (Normal, Abnormal (A, B, C, D)). In this case, two important points were observed: (i) the reflectance varies in a random manner with respect to the caries; and (ii) the reflectance of the signal pertaining to the dentine is higher than that of the enamel. The probable reason for these results is that enamel (which comprises rows of hydroxyapatite) is harder than dentine (which is made up mineralized connective tissues).

**Figure 4:**
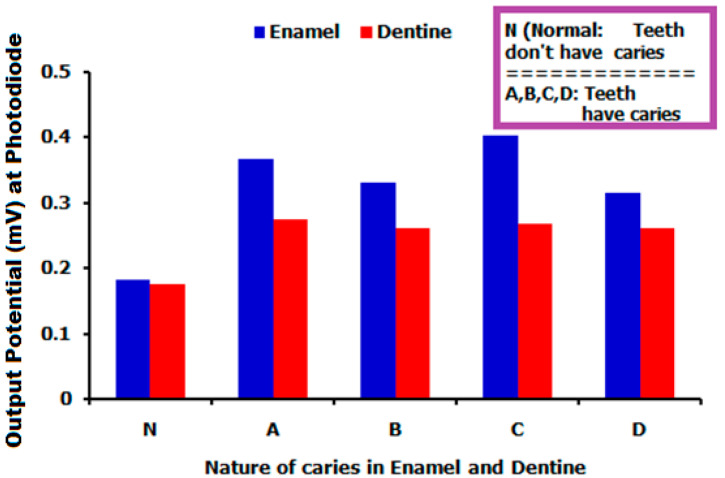
Output potential with respect to the enamel and dentine teeth.

After obtaining the reflectance, the potential appearing at the photodiode was found using Equation (8), which is indicated in Figure 4.

In Figure 4, the nature of enamel and dentine teeth is chosen along the horizontal axis whereas potential appearing at the photodiode is taken along the vertical axis. From the above Figure 4, interesting results are found. For example the output potential for normal teeth (enamel and dentine that does not have any caries) is around 1.8 mV for both cases. However potentials corresponding to the caries are more than 2.6 mV in the case of dentine and more than 3.1 mV in the case of enamel. Apart from this, it is also seen that the output potential for caries in enamel is always more than dentine. So, it is inferred that the nature of the teeth can be investigated by knowing the output voltage collected at the photodetector.

## 6. Conclusions

A one-dimensional photonic structure was employed in the current research to obtain the statistics of the teeth pertaining to the caries at the signal of 1.8 THz frequency. The detailed fabrication feasibility of the glass-based one-dimensional structure and methods of sample collection were also discussed. The reflectance of the signal from the proposed structure played a key role in envisaging the status of the teeth. Moreover the packing of the suggested device comprises the source, the specimen containing one-dimensional structure, and the photodetector. Interesting results were found using the photodetector. For example; the output potential of the reflected signal was 0.175 mV for normal dentine and 0.18 mV for normal enamel teeth. However potentials were 0.332 mV, 0.403 mV and 0.316 mV for enamel caries A, B, C and D respectively. Similarly, potentials were 0.262 mV, 0.268 mV and 0.316 mV for enamel caries A, B, C and D, respectively. Finally, it was inferred that the nature of teeth pertaining to the caries in the enamel and dentine teeth can be investigated by understanding the amount of potential at the output side.

As far as shortcomings of the present work is concerned, the packing system of the source, structure and photodiode is a limitation. A proper packing system with the help of a suitable packing technology could be a future research aim.

## Figures and Tables

**Table 1:** Refractive indices of cell with respect to the deformation.

Specimen	Deformation Size (µm)	Refractive Indices
Normal Enamels	Normal	2.2
Abnormal Enamels	A	3.2
	B	3.0
	C	3.4
	D	2.9
Normal Dentine	Normal	1.9
Abnormal Dentine	A	2.6
	B	2.5
	C	2.8
	D	2.65

**Table 2:** Reflectance of signal with respect to the deformation.

Specimen	Deformation Size (µm)	Reflectance_Enamel_	Reflectance_Dentine_
Normal	Normal	0.02438	0.0235
Abnormal	A	0.04911	0.03682
	B	0.04456	0.03515
	C	0.05417	0.03597
	D	0.04246	0.03514

## Data Availability

All data are available within the manuscript.
